# Induction of Biologically Active Flavonoids in Cell Cultures of *Morus nigra* and Testing their Hypoglycemic Efficacy

**DOI:** 10.3797/scipharm.1101-15

**Published:** 2011-10-03

**Authors:** Ahmed M. A. Abd El-Mawla, Khaled M. Mohamed, Ashraf M. Mostafa

**Affiliations:** 1 Department of Pharmacognosy, Faculty of Pharmacy, Assiut University, Assiut 71526, Egypt; 2 Department of Pharmacognosy, Faculty of Pharmacy, Taif University, Taif 21974, Saudi Arabia; 3 Department of Anatomy and Histology, Faculty of Medicine and Medical sciences, Taif University, Taif 21974, Saudi Arabia

**Keywords:** *Morus nigra*, MS medium, Methyl jasmonate, Flavonoids, Streptozotocin

## Abstract

The antidiabetic activity of both leaves and MJ-treated cell cultures of *Morus nigra* was evaluated after their oral administration to streptozotocin-induced diabetic rats. The antidiabetic activity of extracts from leaves given to streptozotocin (STZ)-diabetic rats for 10 days increased with increasing doses of leaves extract up to 500 mg/kg/day. The administration of 500 mg/kg/day of leaves extract reduced the concentration of glucose from 370 ± 7.31 mg/dl (control) to 154 ± 6.27 mg/dl, and a significant increase in the insulin level from 11.3 ± 0.31 μU/ml (control) to 14.6 ± 0.43 μU/ml was recorded. Cell suspension cultures were established from the young leaves of *Morus nigra* cultivated on modified MS medium supplemented with 2.0 mg/l 1-naphthaleneacetic acid (NAA), 0.2 mg/l 6-(furfurylamino)purine (kinetin). The changes in cell weight and flavonoid content were monitored between day zero and 12. The linear increase in fresh weight was found to be parallel to flavonoids production. Cell cultures treated with 100 μM methyl jasmonate for 24 hours showed a noticeable increase in level of flavonoids and significant and more effective hypoglycemic activity than that for extract from leaves. The major flavonoids were isolated by TLC and HPLC and identified as rutin, quercetin, Morusin and cyclomorusin by co-chromatography and mass spectrometry in comparison to samples of authentic reference compounds.

## Introduction

Traditional herbal medicine has been used for centuries to treat various chronic diseases. There has been some anecdotal evidence to suggest that some of these medicines could be used in treating patients with diabetes and that they may be quite effective in improving the glycemic control in these patients [1–3]. Recently, there has been a growing body of evidence from several studies both in human subjects and animal models with diabetes that such herbal treatment is both safe and effective [4].

Substantial efforts have been made in recent years to identify both natural and synthetic antidiabetics. The screening of more effective and safe hypoglycemic agents has continued to be an important area. Furthermore, after the recommendations of WHO on diabetes mellitus [5], investigation on hypoglycemic agents from medicinal plants has become more essential.

Evidence has shown that *Morus* species has been used traditionally as an antidiabetic herbal medication [3 6–8]. The mulberry belongs to the genus *Morus* of the family Moraceae. There are 24 species of *Morus* and one subspecies, with at least 100 known varieties. The mulberry is found from temperate to subtropical regions of the Northern hemisphere to the tropics of the Southern hemisphere, and they can grow in a wide range of climatic, topographical and soil conditions. These are widely spread throughout all regions from the tropics to the sub-arctic and from sea level to altitudes as high as 4000 m [9, 10]. Biologically active secondary metabolites such as alkaloids, flavonoids, glycoproteins, benzofurans etc. have been isolated and identified from different *Morus* species [3, 11–15].

*Morus nigra* L., belonging to the Moraceae family, is a deciduous tree widely cultivated in Europe and West Asia. It has a long history of medicinal use in Chinese medicine, as a remedy for many kinds of diseases. Phenolic constituents of *Morus nigra* fruits were evaluated and compared with the fruits of another species of *Morus* [16]. The chemical constituents of *Morus nigra* bark have been studied; in addition, three new compounds including two flavonoids and a new 2-phenylbenzofuran were isolated and identified [17, 18]. Micropropagation of *Morus nigra* L. from shoot tip and nodal explants of mature trees was studied [19]. In addition, chalcone dimethylallyltransferase from *Morus nigra* cell cultures was studied with respect to substrate specificity [20].

In the present work, we induced formation of biologically active flavonoids in cell cultures of *Morus nigra* and tested their hypoglycemic efficacy. In addition, the hypoglycemic activity of MJ-treated cell cultures was compared to that of leaves of *Morus nigra*.

## Results and Discussion

### Cell culture growth and flavonoid accumulation

**Fig. 1:**
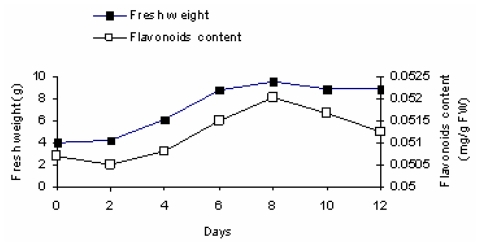
Changes in fresh weight and flavonoids content of *Morus nigra* cell cultures. The data are mean values of two independent experiments.

Cell cultures of *Morus nigra* were derived from leaf explants. A linear increase in fresh weight was observed between day 1 and day 7 after inoculation of cells into fresh MS medium. The major flavonoids were separated by TLC using CHCl_3_- MeOH- H_2_O (8:2:0.5) as solving system. Further purification of the isolated compounds was achieved by HPLC. The flavonoids were identified in comparison to authentic reference compounds. HPLC analysis of flavonoids content of untreated cell suspension cultures of *Morus nigra* revealed a little linear increase in flavonoids content between day 2 and day 8, which paralleled cell growth (Fig. 1).

### Effect of MJ on flavonoids accumulation

Methyl jasmonate is involved in signal transduction and induces the transcription of biosynthetic enzymes involved in the formation of defence compounds in plants [21]. Since MJ can induce the formation of secondary metabolites in other systems, we have tried to induce the accumulation of flavonoids in cell suspension cultures of *Morus nigra* by treating the inoculated cells with MJ.

The cell suspension cultures of *Morus nigra* (4-day-old cultures) were treated with 100 μM MJ and incubated for 24 h. The flavonoids were extracted and determined quantitatively by HPLC. Estimation of total major flavonoids was done by peak area calculation in relation to the peak area of an aliquot of a standard (morusin) solution. MJ at a concentration of 100 μM induced the accumulation of total flavonoids about 12-fold and 7-fold as compared to the untreated cells (control) and leaves respectively (Table 1).

**Fig. 2:**
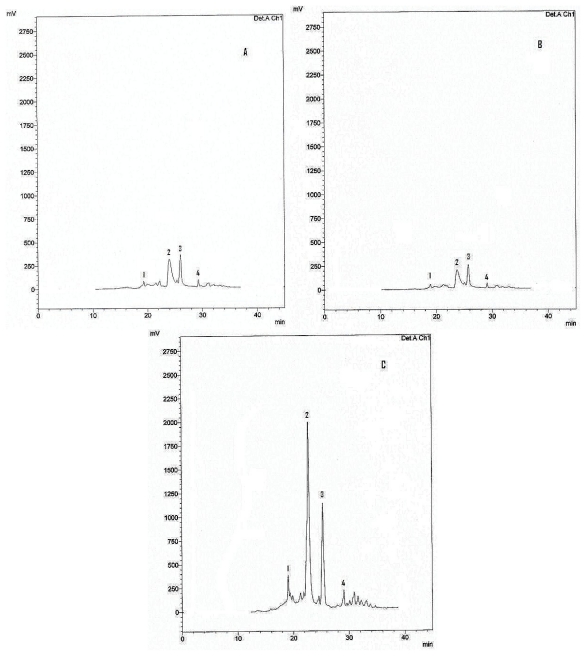
HPLC chromatograms of *Morus nig*ra; A: Extract from leaves, B: Extract from control cell cultures (untreated), C: Extract from cell cultures treated with 100 μM MJ.

The four major flavonoids were isolated by preparative TLC and HPLC and identified as rutin (**1**), quercetin (**2**) morusin (**3**) and cyclomorusin (**4**), by co-chromatography (HPLC and TLC) and mass spectrometry in comparison to samples of authentic reference compounds. The HPLC chromatograms reveal a significant increase in both quercetin and morusin than others (Fig. 2).

### Antidiabetic activity

The antidiabetic activity of extracts from leaves given to streptozotocin (STZ)-diabetic rats for 10 days increased with increasing doses of leaves extract up to 500 mg/kg/day. A dose of 500 mg/kg/day of leaves extract reduced the concentration of glucose from 370 ± 7.31 mg/dl (control) to 154 ± 6.27 mg/dl and a significant increase in the insulin level from 11.3 ± 0.31 μU/ml (control) to 14.6 ± 0.43 μU/ml was observed (Table 2).

The hypoglycemic activity of cultured cells treated with MJ for 24 h was found to be significant and more effective than that of leaf extract. Cells treated with MJ reduced the amount of the glucose from 370 ± 7.31 mg/dl (control) to 145 ± 6.30 mg/dl and significantly increased the insulin level from 11.3 ± 0.31 μU/ml (control) to 15.2 ± 0.32 μU/ml (Table 2). It could be considered that the significant hypoglycemic action of cell suspension cultures is related to additive action of flavonoids induced by MJ treatment. This synergistic action agrees with reported data [6, 7] which proved the hypoglycemic action of *Morus* root bark to be due to synergistic or additive action of moranoline (1-deoxynojirimycin), morans (glycopeptides), hydrophobic flavonoids (flavones and flavanones) and 2-arylbenzofurans.

## Experimental

### General procedure

TLC was performed on silica gel 60 F_254_-coated aluminum sheets (Merck, Darmstadt, Germany). Spots were visualized by spraying with 1% w/v aluminum chloride.

EI-MS was carried out on JEOL JMS 600 Hz (Japan).

HPLC analysis was carried on L-6200A intelligent pump and L-4000 UV detector (Merck, Germany).

The insulin ELIAS was performed on Anthos Labtec Instrument; 400–700 nm wavelength (Austria) and Anthos Fluido 2 Microplate Washer, Biochrome Ltd, Cambridge, UK.

### Plant material

*Morus nigra* plant was collected in the flowering stage from Taif region, Saudi Arabia. Identification of the plant was confirmed by Prof. Hosseny A. Mossallam, Faculty of Science, Taif University, Taif, Saudi Arabia.

### Chemicals and kits

All the media components were purchased from E-Merck (Darmstadt, Germany). The authentic flavonoids; Morusin, cyclomorusin, quercetin and quercetin-3-O-rutinoside (Rutin) were obtained from Chemistry Dept. Faculty of Sciences, Taif University, Taif, Saudi Arabia. Streptozotocin and Glucose (GO) assay kit were purchased from Sigma-Aldrich (Germany). GenWay Insulin-ELISA kit was purchased from GenWay Biotech, San Diego, USA.

### Establishment and maintenance of cell suspension cultures

Leaves of *Morus nigra* were used as explants source. After rinsing in water, explants were sterilized with 70% ethanol for few seconds, immersed in 0.1% mercuric chloride solution for 15 min and washed three times with sterile distilled water. The sterilized leaves were cultivated on 50 ml solid MS medium [22], containing 2.0 mg/l 1-naphthaleneacetic acid (NAA), 0.2 mg/l 6-(furfurylamino)purine (kinetin), 4 g/l phyta-gel and 30 g/l sucrose at pH 5.75 and 25 ±2 °C in the dark.

The resulting callus tissues were subcultured at 3-week intervals. Cell suspension cultures were established by transferring 4 g of callus in 50 ml liquid MS medium (without phyta gel) with the same growth regulator supplementation. Cultures were shaken in 300-ml Erlenmeyer flasks at 100 r.p.m. and 25 ±2 °C in the dark. Cell suspension cultures were subcultured every 7–10 days at the end of the exponential growth phase.

### Growth curve

Ten flasks with fresh liquid MS medium (50 ml) were prepared and inoculated with 4 g of cultured cells of *Morus nigra* (day zero). Fresh weight (FW) and flavonoids content were determined from day zero to day 12 at two-days-intervals. Cultured cells were harvested by vacuum filtration.

### Effect of methyl jasmonate

4 gm (FW) of cultured cells of *Morus nigra* were inoculated in 50 ml MS medium. Cultured cells of *Morus nigra* in exponential phase (4-day-old cultures) were exposed to 100 μM methyl jasmonate (Sigma, Germany) for 24 h. Cultured cells were harvested by vacuum filtration, weighed and kept at −20 °C until the extraction and HPLC analysis. Growth (FW) and flavonoids accumulation in the cultured cells were determined.

### Extraction and phytochemical investigation

Both the leaves and cell cultures (control & treated) of the plant (250 g) were separately extracted by maceration with 70% ethyl alcohol at room temperature. The phytochemical and chromatographic screening of the extracts from both the leaves and the treated cells revealed the presence of flavonoids.

**Fig. 3:**
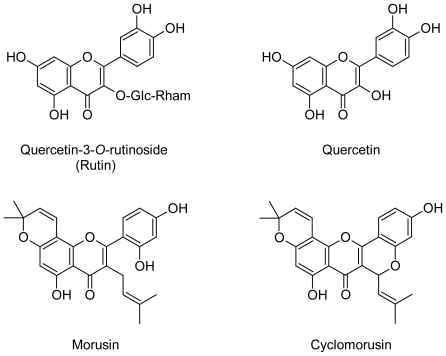
Chemical structure of the isolated flavonoids

TLC screening using CHCl_3_/MeOH/H_2_O (7.5 : 2.5 : 0.5 v/v/v) as a solvent system of treated cells extract indicated that they are richest in flavonoids (AlCl_3_ detection) and revealed several spots, four of them were major. They were isolated by a preparative TLC on silica gel G_60_ F_254_-coated glass sheets (Merck, Germany). Further purification of the isolated compounds was achieved by HPLC. This was performed on a reversed phase – (RP)-8 column (Nucleosile^®^ 100-5; 25 cm long, 0.4 cm i.d.; Macherey-Nagel, Dueren, Germany) using water (A) and methanol (B) as the solvents. The following gradient was used: 20% B for 5 min, 20–70% B within 30 min, then isocratic elution at 70% B. The flow rate was 1 ml/min and the detection wavelength set to 360 nm. Their EI-mass spectra agreed with published data for quercetin-3-*O*-rutinoside (rutin), quercetin, Morusin and cyclomorusin (Fig. 3) [15–17]. Furthermore, co-chromatography (TLC & HPLC) with authentic flavonoids was performed.

### Quantitative determination of the flavonoids

One gram of both fresh cells (control & treated) and fresh leaves of *Morus nigra* were separately ground with 7 ml 70% EtOH till a homogenate. The homogenate was filtered and the residue was re-extracted twice with 5 ml 70% EtOH. The combined ethanolic extract was evaporated till dryness. The residue was re-dissolved in 1 ml MeOH and filtered. A serial dilution was made from this solution. The prepared solutions for both fresh cells and leaves of *Morus nigra* were used for quantitative determination of the flavonoids using HPLC as mentioned above in purification of the isolated compounds. Morusin in a concentration of 1 μg/100 μl was used as a reference compound for flavonoids. The quantity of total flavonoids was estimated on the bases of their peak area relative to the area of morusin (0.3 μg) as external standard.

### Animals and induction of diabetes

Male Wister rats weighing 170–260 g were obtained from the experimental animal care centre, King Abdelaziz University, Jeddah, KSA. Induction of diabetes was performed as previously published [3]. The STZ was dissolved in 0.1 ml of citrate buffer (pH 4.5). Animals were made diabetic by injection of a single dose of STZ (60 mg/kg) intra-peritoneally.

STZ-treated rats were given 5% glucose in their drinking water for the first 24 h to counter any initial hypoglycemia. Control animals were similarly injected with vehicle only. On the third day, the animals were checked for the presence of glucose in the urine using enzymatic test strips. The animals were maintained under standard conditions of temperature 24±5 °C and 55±5% relative humidity with a regular 12 h light: 12 h dark cycle and allowed free access to standard laboratory food (Purina Chow) and water. All animals were treated humanely in accordance with the guideline for care of animals as set by WHO.

### Biological study

As mentioned previously in literature [3]; the rats were randomly divided into four groups of 10 animals each. The first group was received citrate buffer alone (control), the second was STZ-diabetic rats, the third was the STZ-diabetic rats fed orally with extract from leaves of *Morus nigra* [100–600 mg/kg/day (one hundred intervals)], and while the fourth was the STZ-diabetic rats fed orally with extract from MJ-treated *Morus nigra* cell cultures [100–600 mg/kg/day (one hundred intervals)] for 10 successive days.

On the eleventh day, the rats were subjected to light ether anaesthesia and killed by cervical dislocation. Trunk blood was collected into heparinised chilled tubes containing sodium fluoride (to inhibit glycolysis). Serum was separated by centrifugation at 4 °C for 15 min at 3000 rpm and stored at −20 °C until determination of serum glucose and serum insulin concentrations.

### Serum analysis

Glucose oxidase [23] and GenWay INS-ELISA [24] were used for estimation of serum blood glucose and serum insulin levels respectively.

### Statistical analysis

Data are expressed as means ± S.E.M. Statistical comparison between different groups were done using one-way analysis of variance (ANOVA) followed by the Tukey–Kramer multiple comparison test, to judge the difference between various groups. Significance was accepted at *P* < 0.05.

## Figures and Tables

**Tab. 1:** Flavonoids concentration in leaves and cell suspension cultures of *Morus nigra*.

Extract	Flavonoids content (mg/g fresh weight), ± SD
Leaves	0.230 ± 0.010
Control cell cultures (untreated)	0.133 ± 0.021
Cell cultures treated with 100 μM MJ	1.610 ± 0.011

* The data are mean values of two independent experiments.

**Tab. 2:** Effect of leaves and treated cells extracts on serum glucose (mg/dl) and serum insulin hormone (μU/ml) in STZ-diabetic treated rats.

	Glucose level (mg/dl) ± SE	Insulin level (μU/ml) ± SE
STZ-diabetic rats	370 ± 7.31*	11.3 ± 0.31
Control (untreated rats)	142 ± 5.22	16.3 ± 0.41
STZ-diabetic rats fed on extract from leaves (mg/kg)		
100	300 ± 7.45*	11.5± 0.43*
200	281 ± 6.34*	11.9 ± 0.62*
300	211 ± 8.33*	12.3 ± 0.74*
400	190 ± 4.11*	12.8 ± 0.51*
500	154 ± 6.27*	14.6 ± 0.43*
600	154.3 ± 5.19*	14.7 ± 0.62*
STZ-diabetic rats fed on extract from MJ-treated cell cultures (mg/kg)		
100	290 ± 5.13*	11.8 ± 0.42*
200	277 ± 6.23*	12.9 ± 0.64*
300	200 ± 8.31*	14.1 ± 0.73*
400	180 ± 4.17*	14.8 ± 0.51*
500	145 ± 6.30*	15.2 ± 0.32*
600	144 ± 7.18*	15.3 ± 0.52*

* Data are expressed as means ± S.E.M.

* *P* < 0.05 between normal and diabetic Controland between diabetic control and both diabetic fed on leaves extract and diabetic fed on treated cells extract.
